# Factors affecting adherence to anti-retroviral therapy among women attending HIV clinic of a tertiary health institution in SouthEastern, Nigeria

**DOI:** 10.4314/ahs.v22i1.54

**Published:** 2022-03

**Authors:** Hope C Opara, Peace N Iheanacho, Blessing Nebo, Justin A Ingwu, Chinenye J Anetekhai, Agnes N Anarado

**Affiliations:** Department of Nursing Sciences, Faculty of Health Sciences and Technology University of Nigeria Enugu Campus

**Keywords:** Adherence, anti-retroviral therapy, factors, women, HIV Clinic, Enugu

## Abstract

**Background:**

Strictly adherence to antiretroviral therapy (ART) is needed to achieve viral suppression. Studies have focused on HIV positive pregnant women's adherence. Factors affecting non-pregnant HIV positive women's adherence has been understudied in Enugu

**Objective:**

The study objective was to identify factors affecting adherence to ART among HIV positive women attending retroviral clinic of a tertiary hospital in Enugu.

**Methods:**

Using a descriptive cross-sectional design, a pre-tested structured questionnaire was used for data collection among 286 HIV positive women aged 18 years and above. Data were analyzed using descriptive statistics of proportions, percentages, and means. Responses with a mean score of ≥2.5 were taken as important factor affecting adherence.

**Results:**

Overall adherence was 56.2%. Participants were considered adherent if they took ≥95% of their prescribed ART. Lack of transport fare (2.69 ±1.36), long-distance to clinic (2.82±1.26), health workers' poor attitude (2.74±1.28), and lack of partners' and parents' support (2.57±1.05) affected adherence negatively while ease in renewing prescription and minimal side effects of drugs enhanced adherence. Enfuvirtide (21.1%) and Lamivudine (17.4%) were drugs that were mostly skipped.

**Conclusions:**

Adherence to ART was low among the women attending the HIV clinic in Enugu. Adherence counseling and education should be provided before ART initiation. Strategies to reduce stigma, increase family support, and improve healthcare providers' attitudes should be employed.

## Introduction

Human Immunodeficiency Virus/Acquired Immune Deficiency Syndrome (HIV/AIDS) has remained a public health problem. The World Health Organization^1^ proposed the use of antiretroviral therapy (ART) to suppress HIV/AIDS and prevent mother to child transmission (MTCT) of HIV. Strict adherence to antiretroviral drugs is needed to achieve a maximal reduction of HIV/AIDS symptoms, ensure the maintenance of sufficient concentrations of antiretroviral agents needed to suppress HIV replication and lower the plasma viral load^2^, ^3^ Although these medications are effective, issues with adherence have prevented many people living with HIV from benefitting from ART because 50% do not take their medications as prescribed 4, 5

About 35.9 million people are currently living with HIV/AIDS worldwide of which 23.5 million are in sub-Sahara Africa6 Nigeria has a current HIV prevalence of 4.1% with about 3.2 million infected with the virus and an estimated 1.6 million eligible for Anti-retroviral drugs^7^. Globally an estimated 1.4 million HIV infected women give birth each year of which 91% of them reside in sub-Saharan Africa8 Using antiretroviral therapy (ART) during and after pregnancy helps to preserve maternal health and prevent mother-to-child transmission of HIV^9^. Expanded access to and uptake of ART during pregnancy has increased dramatically bringing obvious global reductions in mother-to-child HIV transmission (MTCT) 10,11.

The “90-90-90” United Nations agenda launched in 2014 proposed that 90% of all people living with HIV will know their HIV status, 90% of all people with diagnosed HIV infection will receive sustained antiretroviral therapy and 90% of all people receiving ART will have viral suppression by 2020^3^. Studies showed that in Western and Central Africa, HIV testing and treatment coverage are far below the global average12 and a number of patients took less than 95% of antiretroviral therapy^2^. The success of HIV management depends seriously on good adherence; as poor adherence can lead to the development of drug limiting resistance by the virus^13^, ^2^.

Antiretroviral therapy consists of a lifelong complex treatment that often involves varying dosing schedules, dietary restrictions, and adverse side effects which makes patient's adherence inconsistent. Even with more tolerable and simpler regimens currently available the overall rate of adherence to ART still remains suboptimal globally^14^. In low and middle-income countries that have over 90% of the infected population, access to ART has dramatically expanded in the last decade9 However it is estimated that only 47% adults and 23% children who are eligible are accessing treatment and less than half of the patients had 100% adherence^15^.

Medication adherence is defined as the process by which patients take their medications as prescribed by their health care providers. It entails the start of the treatment, the extent to which the patient follows the dosing regimen, and the non-interruption of treatment^16^. Adherence can be measured through methods like Therapeutic Drug Monitoring (TDM), Directly Observed Therapy (DOT), pill counts, and self-report^17^. The self-report method which was used in this study is where the patient or caregiver in the case of children supplies information on how the medication was taken and the doses that were missed. Adherence in this study entails that patients should take up to 95% or more of the antiretroviral drug doses to maintain suppression of viral replication^18^. Different levels of adherence to ART have been reported in different localities. Studies in Tanzania and Ethiopia recorded 84% and 65% adherence to ART respectively 19,2 However in United States, adherence level of 75% was noted among women during pregnancy which reduced to 65% at 6 weeks post-partum 20.

Adherence to ART by patients has continued to be suboptimal^2^ which might be due to multi- factorial causes. Some of such factors reported in the literature include patient-related factors, inconvenient dosing frequency, pill burden, side effects, patient-health-care provider relationships, lack of money for transportation to the hospital, busy schedule, and forgetting^9^, ^21^, ^22^. Any factor that influences adherence to regimen should be a concern to health care providers. These factors and how they impact on adherence behavior of women has been understudied 23. Women culturally take care of family members but may receive little or no attention especially in poorly resourced countries with less empowerment.

Non-adherence is costly and can lead to drug resistance, opportunistic infections, frequent admissions, and increased cost of care resulting in reduced quality of life of patients 7,9. There is a dearth of empirical work from Nigeria especially in the study setting relating directly to adherence to ART among pregnant and non-pregnant HIV positive women of all ages and the influencing factors. Diverse levels of adherence to ART were reported for the different geo-political zones in Nigeria. Values recorded for South-west 24, South-south 21, and South-east zones 25 were 70.8%–92.6%, 49.2%–72.2% and 42.3%–81.4% among all genders.

The question that informed this study was: To what extent do women living with HIV (pregnant and not pregnant) adhere to ART therapy and what factors affect their adherence behavior? This study, therefore, sought to identify factors affecting adherence to anti -retroviral therapy among women attending the HIV clinic of a tertiary health institution in Southeastern, Nigeria. Identifying and addressing these factors will help to develop measures to enhance adherence and improve the quality of life of women living with HIV/AIDS.

## Methods

The study was a descriptive cross-sectional study. Using a Power Analysis Formula for finite population, a sample of 286 women living with HIV/AIDS was enlisted from a population of 1,118 HIV positive women that were registered and receiving ART from the HIV clinic of the University of Nigeria Teaching Hospital Enugu. The setting was chosen for the study because it has a comprehensive HIV Clinic that serves as a referral center for Southeast Nigeria and environs. Ethical clearance was obtained from the Health Research and Ethics Committee of the study center (NHREC/05/01/20083-FWA00002458-IRB00002323) while the administrative permit was obtained duly from appropriate authorities. Informed consent was obtained from the patients after a detailed explanation of the study. Confidentiality and anonymity of information were ensured.

A pre-tested, structured researcher- developed questionnaire was used to collect information about respondents' characteristics; the level of adherence to ART, socio-economic, psychological, and regimen factors affecting adherence to ART. In measuring the perceived factors affecting their adherence, responses were scaled and scored as: Strongly agree (SA=4), Agree (A=3), Disagree (D=2) Strongly disagree (SD=1). The questionnaire was interviewer-administered to accommodate literate and illiterate women. The researcher collected data during the four HIV clinic days per week from August to October. 2019. All consenting women present at the HIV and PMTCT clinics during the study period were recruited and interviewed, till the sample size was completed. Data were analyzed with the Statistical Package for the Social Sciences version 23 using descriptive statistics of proportions, percentages, and means. Factors with a weighted mean score of ≥2.5 are seen as having an influence on adherence behavior of the respondents.

## Results

### Respondents' characteristics (Table 1)

**Table 1:** Socio-Demographic Distribution of the Respondents n = 265

Socio-demographic characteristics	Frequency	Percentage (%)
**Age**		
18 – 29	68	25.7
30 – 41	115	43.4
42 and Above	82	30.9
**Educational Qualification**		
None	47	17.7
Primary Education	96	36.2
Secondary Education	60	22.6
Tertiary Education	62	23.4
**Religion**		
Christianity	232	87.5
Muslim	33	12.5
**Marital Status**		
Single	48	18.1
Married	148	55.8
Divorced	14	5.3
Widowed	55	20.8
**Occupation**		
Civil Servant	34	12.8
House Wife	27	10.2
Self-Employed	175	66.0
Unemployed	29	11
**Ethnicity**		
Igbo	251	94.7
Hausa	14	5.3

Table 1 showed that more women (43.4%) were within the 30 – 39 years age category, 96 (36.2%) had only primary education while 232 (87.5%) were Christians. A little more than half of the respondents (148 =55.8%) were married, 175 (66%) were self-employed and 251 (94.7%) were of the Igbo tribe.

### Level of adherence to antiretroviral therapy among women (Table 2)

**Table 2:** Level of Adherence to Antiretroviral Therapy (ART) n = 265

Items	Options	Frequency(Percentage)
How long have you been on ART?	Below 1 year	42 15.8%
	Above 5 year	223 84.2%
Which of the following drugs are	Enfuviritide	15 5.7%
you taking	Dolutegravir	6 2.3%
	Tenofevir	48 18.1%
	Lamivudine	61 23%
	Nevirapine	54 20.4%
	None	48 18.1%
	All	26 9.8%
	Can't remember	7 2.6%
Did you miss taking any of your	Yes	124 46.8%
medication in the past one week?	No	135 50.9%
	Can't remember	6 2.3%
How many days did you miss your	2days	51 41.1%
drug in the past one week?	4days	23 18.9%
	6days	7 5.3%
	Can't remember	43 34.7%
Which of the drugs did you miss	Enfuviritide	56 21.1%
taking in the past one week?	Dolutegravir	27 10.2%
	Tenofevir	27 10.2%
	Lamivudine	46 17.4%
	Nevirapine	13 4.9%
	Can't remember	96 36.2%

Table 2 showed that majority of the women (223=84.2%) have been receiving Antiretroviral Therapy for more than 5years, 61 (23%) were placed on Lamivudine, 48 (18.1%) on Tenofovir, 54(20.4%) were on Nevirapine, 15 (5.7%) on Enfuviritide and 26 (9.8%) on combination therapy of (Tenofovir, Lamivudine, Nevirapine, Enfuviritide, and Dolutegravir). Only 164 (56.2%) took their medication completely in the previous week to the study, while 135 (50.9%) could not take medication completely. Out of those who could not adhere, 109 (41.1%) reportedly defaulted for 2days in the week, 50(18.9%) for 4days and 92 (34.7%) were non adherent for 6days in the week.

### Socio-economic factors affecting adherence to ART (Table 3)

**Table 3:** Socio-economic factors affecting adherence to ART n = 265

Items	SA	A	D	SD		StD
Lack of transport fare to ART clinic to renew prescription	89(33.6%)	14(5.3%)	33(12.5%)	114(43%)	*2.69	1.36
Long distance from home to ART clinic to collect new supply of drugs	75(28.3%)	14(5.3%)	60(22.6%)	116(43.8%)	*2.82	1.26
Lack of support from government, non-governmental organization to subsidize cost of drugs	90(34%)	81(30.6%)	94(35.5%)	-	2.02	0.84
Good communication and relationship with healthcare providers	205(77.4%)	33(12.5%)	13(4.9%)	145.3%)	1.38	0.81
Poor attitude of healthcare workers is discouraging	77(29.1%)	28(10.6%)	47(17.7%)	113(42.6%)	*2.74	1.28
I don't find it difficult to renew my prescription	20(7.5%)	28(10.6%)	130(49.1%)	87(32.8%)	*3.07	0.86
Lack of support from partner and parents	60(22.%6)	49(18.5%)	102(38.5%)	54(20.4%)	*2.57	1.05
**Grand Means**					**2.47**	**1.07**

Decision rule: weighted mean score of ≥2.5 shows significant influence on adherence

Table 3 showed that lack of transport fare to ART clinic to renew prescription (2.69±1.36), a long-distance from home to ART clinic (2.82±1.26), discouraging/ poor attitude of healthcare workers (2.74±1.28), and lack of support from parents and partners (2.57±1.05) affected adherence negatively while for those who adhered, ease of renewing prescription (3.07±0.86) enhanced adherence.

### Psychological factors affecting adherence to ART (Table 4)

**Table 4:** Psychological factors affecting adherence to ART n = 265

Items	SA	A	D	SD		Std
Sometimes I feel depressed about my HIV status making me not to take my drugs	148(55%)	6(2.3%)	41(15.5%)	70(26.4%)	2.13	1.33
Non disclosure to my partner or family member makes me hide or avoid my drugs	96(36.2%)	75(28.3%)	67(25.3%)	27(10.2%)	2.09	1.01
Lack of support from family discourages me	89(33.6%)	75(28.3%)	68(25.7%)	33(12.5%)	2.17	1.03
Stigma of been identified as HIV positive person	123(46.4%)	108(40.8%)	7(2.6%)	7(2.6%)	1.58	0.69
I have good support from family so I take my drugs	121(45.7%)	83(31.3%)	55(20.8%)	6(2.3%)	1.80	0.85
Support from others in the group makes me bold	155(58.5%)	95(35.8%)	15(5.7%)	-	1.47	0.60
The need to take the drug for lifetime is tiring	108(40.8%)	81(30.6%)	28(10.6%)	48(18.1%)	2.06	1.11
I have accepted the long duration of treatment so I take my drugs	94(35.5%)	130(49.1%)	27(10.2%)	14(5.3%)	1.85	0.81
**Grand Mean**					**1.89**	**0.93**

Decision rule: weighted mean score of ≥2.5 shows significant influence on adherence

Psychological factors were not seen as important among the factors that affect adherence behavior with weighted mean values 2.5 as seen in table 4. However, some respondents did report that some psychological factors such as feeling depressed about HIV status, non-disclosure to partner or family member (which made patient hide or avoid drugs), fear of stigmatization and having to take the drug for a lifetime had some effects on their adherence behavior.

### Regimen factors affecting adherence to ART (Table 5)

Table 5 showed that the only regimen factor that affected adherence to ART was experiencing minimal side effects by the patients (2.52±0.99). Other factors like forgetfulness, pill burden, and having to take the drug at the same time of the day (mean < 2.5) were not reported as important in affecting their adherence to ART. This showed that having minimal side effects from the drugs makes the patient take their drugs as prescribed.

## Discussion

Patients' adherence to ART in this study was 56.2%. This is close to the findings of previous studies 21, 26, and20 that recorded adherence of 50.4%, 65%, and 69% respectively. These levels of adherence are rather low and a far cry from UN projections for 20203. Incidentally, these studies were all done in Africa. A much lower adherence level of 32.7% has also been reported elsewhere in Nigeria^12^. In contrast, there were other studies 7, 18 that had higher adherence levels of 89.2% and, 86.7% among only pregnant women accessing treatment at PMTCT clinics. This variation in adherence among pregnant women may be due to the greater effort put into the care of pregnant women and the desire to be healthy to care for their born and unborn children.

The low adherence to ART recorded in this study may be due to poor understanding of pre-counseling information on drugs, dosage, and consequences of non-adherence before initiation of ART considering that up to 36.2% had only primary education. Another study corroborated this assertion that low educational level deters adherence to ART as respondents may not understand the importance of strict adherence to ART. In their study 52.2% of the respondents had primary education and 28.3% could not read or write which impacted adherence negatively^27^.Women that may be hiding their medication from their families or spouses due to non-disclosure and fear of stigmatization may have difficulty with adherence^15^, ^2^.

Socio-economic factors identified that affected adherence to ART in this study were those related to access to services, such as long-distance from home and lack of transport fare to ART clinic. The health facility is located outside the Enugu metropolis making the cost of transport a burden for the respondents who would need to board more than one vehicle to reach the center. In a situation where the majority of the women were either self-employed (66%), housewives (15.7%) or unemployed (11%) in a nation rife with inflation and political instability, it is understandable why the issue of transport fare was an important one^28^. Previous studies in other parts of Africa also 26–27 recorded difficulty in traveling for ART appointments by respondents, lack of financial support, and cost of treatment as barriers to adherence. However, many respondents indicated that they did not find it difficult to refill their prescriptions and this was a positive factor for them. This may be because their prescription lasts for 3 months before another refill which was enough time for respondents to save money and plan for a clinic appointment.

Poor attitude of health care workers reportedly affected adherence to ART in this study. Health facilities are generally poorly staffed in many resource-poor nations, as was also observed in the study setting. The associated increased workload on available staff may lead to unmet expectations and inadequate attention given to the respondents. Patients' satisfaction involves the patient's conclusion and reactions to how they view the healthcare environment, healthcare provider's attitude, promptness of care received and waiting time before and during consultation^29^. If people living with HIV/AIDS rate the attitude of care providers as poor, which implies dissatisfaction, it may affect their coming for a clinic visit to refill their prescriptions. This is in agreement with the conclusions of other studies 26–27 who indicated that poor relationships with health workers and lack of support visits from health workers affected ART adherence.

Other findings of this study showed that a lack of support from partners and parents affected adherence. Fear of stigmatization and rejection by the spouse or family members have hindered disclosing HIV status^9^. Women that may not have disclosed their status to family members and partners may not get the appropriate support needed. Other studies^26^–^27^, ^30^ recorded the same factors as barriers to adherence to ART in their studies. A study in Ebonyi State, Nigeria noted that encouraging partner support made their patients to adhere better to ART 7. The respondents whose spouses also tested positive and attend the clinic together may have better support. It is worthy of note, however, that some respondents in this study did not have difficulty with social support. This may be due to the strong existing support groups created in the health facility by the facilitators that lend support to respondents.

Findings also revealed that side effects of drugs, forgetfulness, long duration of ART, and pill burden were not important factors affecting respondents' adherence behavior. This may mean that majority of them may have adjusted to the drugs, having been taking the drugs for more than 5years. Having two or more drugs combined into one pill may also have reduced pill burden thereby increasing adherence. The assumption that patients use reminders like alarms may have helped reduce forgetfulness. Studies in Nepal^18^ and South-south Nigeria^13^ reported the use of text messages and calls as reminders to enhance adherence. On the other hand, other studies identified forgetfulness, 7,2,30 side effects and pill burden^18^, ^20^ afactors in their studies that affected patients' adherence negatively. Factors affecting adherence to the regimen is therefore multifaceted and should be addressed holistically.

## Implications of the Study for Nursing

The findings of the study raise issues for the adequacy of the counseling and psycho-education provided by health care workers especially, nurses, before ART initiation. The need for a deliberate effort at effective communication and therapeutic patient-provider relationship can never be overemphasized and therefore, should be improved. The need has also been raised for ART Support groups to move closer to the people in their localities, probably using their health posts for improved follow up and to reduce travel time and cost. Use of reminders like text messages and phone calls, and advocacy to subsidize the cost of treatment should be employed.

## Limitations to study

The study involved women living with HIV at the only comprehensive HIV clinic in Enugu with free or highly subsidized medication. Evidently, not all HIV positive women in the region access the services. Therefore, studying only those who obtain treatment at this center may limit the generalizability of the study findings. Studying a larger population of women from State and faith-based hospitals which are quite many in the area of study may shed more light on understanding the dynamics of adherence and even service utilization as a whole.

## Conclusion

The level of adherence to ART was low. Lack of transport fare, long distance of ART clinic from home, lack of support from partner and parents and poor attitude of healthcare providers were identified factors affecting adherence to ART among women in Enugu.
